# Fitting consistent knowledge into the planning process: An integrated database on adaptation and mitigation measures in Europe

**DOI:** 10.1016/j.dib.2024.110580

**Published:** 2024-06-02

**Authors:** Gerard Martínez Görbig, Johannes Flacke, Matthew Keller, Noah Pflugradt, Richard Sliuzas, Diana Reckien

**Affiliations:** a Faculty of Geo-Information Science and Earth Observation (ITC), University of Twente, Enschede 7522 NH, the Netherlands; b Institute of Energy and Climate Research—Techno-Economic Systems Analysis (IEK-3), Forschungszentrum Jülich GmbH, Wilhelm-Johnen-Straße, Jülich 52428, Germany

**Keywords:** Climate action, Adaptation, Mitigation, Climate planning, Europe

## Abstract

Climate action is far from meeting the internationally agreed adaptation and mitigation goals. Even though climate action planning has increased since the Paris Agreement in 2015, the implementation rate of those plans remains low. Climate planning literature claims that accounting for long-term planning and implementation times, accurately estimating costs, identifying synergies and trade-offs between measures, or considering justice and equity issues might increase the quality of climate plans and facilitate the further implementation of climate actions.

Also, there is no uniform way of responding to the climate crisis. Existing climate action databases typically focus on a particular type of response, sector, hazard, or type. In parallel, national governments and international initiatives provide tools and guidelines to facilitate the development of climate action plans. However, the primary climate action recording and monitoring initiatives and projects do not share the same framework as those tools, resulting in a lost opportunity to improve climate actions' knowledge transferability.

Thus, we reviewed nine existing databases of adaptation and five mitigation databases, comprising a total of 7.130 adaptation actions and 11.409 mitigation actions, and detected a lack of alignment with climate planning practices and claims. Furthermore, we revealed a lack of coherency regarding the level of abstraction of climate actions and their role in the implementation process. Not all climate actions are meant to operate similarly from a planning perspective: while some had a direct outcome on the target indicators, others are thought to facilitate their implementation.

Ultimately, we created a new integrated database of adaptation and mitigation measures in Europe, focusing exclusively on climate planning and implementation practices. First, we identified specific and transferable mitigation and adaptation measures and instruments through an originally designed decision tree. Second, we harmonised the collection of climate actions in a unique framework based on one of the biggest climate planning initiatives: the Sustainable and Energy Climate Action Plans by the Covenant of Mayors. Our integrated database of adaptation and mitigation measures (1) classifies and relates the different types of climate actions; (2) provides data that may improve the quality of climate plans and facilitate implementation; (3) allows a better perspective of systematic problems by identifying potential synergies and trade-offs; and (4) defines and characterises measures using a framework that draws on actual practice. The database compiles a total of 191 adaptation measures, 188 mitigation measures, and 97 measures that account for each, and a total of 609 associated instruments. For monitoring their outcomes, 93 Sustainable Development Goals relevant indicators are included.

Specifications Table

**Table:** 

Subject	Planning and development
Specific subject area	The data falls into climate planning, climate change mitigation and adaptation.
Data format	Analyzed, Filtered
Type of data	Table
Data collection	We analysed thirteen existing climate action documentation initiatives, climate planning documents, and climate action databases, covering 7.130 adaptation and 11.409 mitigation actions. To ensure the significance of the sample, we set the following criteria: (1) climate actions must be planned or implemented within the European context; (2) climate actions are defined in sufficient detail; (3) climate actions come from on-ground research.
Data source location	Sources used to obtain climate actions data were: 1. Carbon Disclosure Project: 1.406 mitigation actions and 977 adaptation actions. −2022.CitiesAdaptationActions dataset. −2022.CitiesEmissionsReductionActions dataset. Link: https://data.cdp.net/browse
	2. 6th Assessment Report by IPCC: 138 mitigation actions and 697 adaptation actions. -No specific list of actions. Searched in the report for the strings “Adaptation option*”, “Adaptation measure*”, “Mitigation option*”, “Mitigation measure*”. Link: https://www.ipcc.ch/ 3. Climate-ADAPT. 59 adaptation actions. Link: https://climate-adapt.eea.europa.eu/#t-database 4. RESIN. 100 adaptation actions. Link: https://resin-aol.tecnalia.com/apps/adaptation/v4/ 5. RESCCUE. 178 adaptation actions. Link: https://adaptationstrategies.resccue.eu/measures 6. CLARITY. 18 adaptation actions. Link: https://clarity-h2020.eu/content/downloads 7. European Environmental Agency Mitigation Measures and Policies Database. 498 mitigation actions, after filtering by only individual measures. Link: http://pam.apps.eea.europa.eu/ 8. Sustainable Mobility for All Catalogue of Policy Measures 2.0. 194 mitigation actions. Link: https://www.sum4all.org/key-products/catalogue-policy-measures-cpm 9. TEG Taxonomy Database. 72 mitigation actions. Link: https://finance.ec.europa.eu/system/files/2020–03/200309-sustainable-finance-teg-final-report-taxonomy_en.pdf 10. ENSU 2022 Sufficiency Policy Database. 254 actions. Link: https://energysufficiency.de/en/policy-database-en/ 11. GcoM – MyCovenant, 4th Release – March 2023. 13.950 adaptation and mitigation actions (with a detailed description) in Europe. 5.100 adaptation actions and 9.564 mitigation actions (only the ones that take place in European context). Link: https://data.jrc.ec.europa.eu/dataset/b425918f-53a1-495c-8619-cd370c302eb0
Data accessibility	Repository name: Zenodo Data identification number: 10.5281/zenodo.8248587 Direct URL to data: https://zenodo.org/record/8248587 Instructions for accessing these data: Read the _ReadMe.pdf file. If possible, download the .xlsm files. If Macros are not allowed due to the user accessibility rights and restrictions, please, download the .zip file and follow the instructions in the _ReadMe.pdf file.

## 1 Value of the Data

• **Focus on implementation:** climate actions in the database are segregated according to their role in the planning process towards implementation. Measures are climate actions that directly impact their environment, can be implemented, and respond to a mitigation or adaptation goal [1,2] – installing solar panels. On the other hand, instruments facilitate the implementation of measures and do not have an outcome directly related to climate mitigation or adaptation [1,2] – subsidies to households for installing solar panels.
• **Coherency of climate actions:** We provide a harmonised dataset of mitigation and adaptation measures at a similar abstraction level. Thus, all are detailed enough to ensure proper transferability to climate plans. Following the previous framework, relevant instruments and indicators are also provided per measure.
• **Useful data from the planning perspective:** The database offers planners not only a set of data, but a structure to follow to boost implementation. An appropriate planning scale [3,4], the proper consideration of implementation times and long-term planning [3,4], and the consideration of justice [[5], [18]] increase the feasibility of climate actions. Identifying synergies and trade-offs is beneficial for implementing climate action and increasing their effectiveness [3,4,6], but they are not typically included in the consulted sources.
• **Transferable climate planning practices framework:** Even if implementation depend on local and regional factors, trusting systematic approaches offers a solution to speed up climate action. The Covenant of Mayors is one of the biggest networks, with more than 11.000 signatories committed to developing a Sustainable Energy Climate Action Plan (SECAP). Thus, we aligned our data with their SECAPs template, thereby offering climate planners directly transferable data for developing climate plans.
• **Integrated approach:** The database integrates mitigation and adaptation measures and identifies measures with outcomes in both response types. Moreover, it identifies synergies within sectors and hazards, provides hints about synergies with SDGs through indicators, and attaches instruments to each measure. As the data comes from relevant European initiatives based on on-ground research, it provides a good overview of how the climate response is in Europe.
• **Replicable methodology:** Climate action is highly context-dependent [2,3,4]. Moreover, new technologies and measures to cope with climate change mitigation and adaptation are being constantly developed. Thus, our work relies not only on data compilation but on a replicable and transparent method, allowing us to easily expand the database with new measures and instruments.

## 2 Data Description

### 2.1 Relational database

**Fig. 1:**
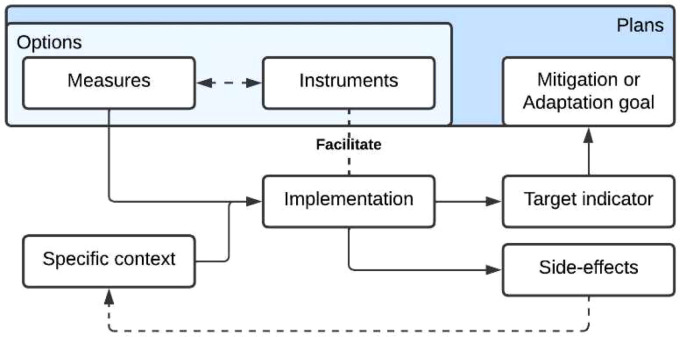
The role of the different climate action types in the planning and implementation process. Source: Authors.

The database [19] is compiled in a single Microsoft Excel file comprising four data sheets which are described in Table 1. The four datasheets altogether compose a relational database. Thus, each datasheet is related to the other through specific relationships based on the climate planning framework described in Fig. 1. The code that allows the database to function can be found in Table S2 of the Supplementary Materials.

**Table 1:** Generic description of the database. Source: Author.

Sheet	Name	Brief description
Measures datasheet	_Measures	It contains an original compilation of adaptation and mitigation measures, structured in a matrix of 477 rows and 18 variables.
Instruments datasheet	_Instruments	It contains 609 instruments, characterised by 8 different variables.
Indicators datasheet	_SOIs	It contains 93 Sustainable Development Goals Oriented Indicators (SOIs), extracted from the work of Ibáñez Iralde & Pascual, 2022 [7]
User interface sheet	_Display	It contains a navigation tool that allows one to easily explore the database and the connections between the different datasheets through a simple interface.

From a climate planning perspective, climate actions are not equal in function and outcome. Plans are composed of a series of climate actions and their targeted goals. Measures are climate actions that can be implemented and directly impact their environment by targeting a mitigation or adaptation goal and modifying the correspondent indicator [1,2]. More specifically, instruments aim to facilitate the implementation of measures and modify their implementation conditions [1,2]. Measures and instruments can sometimes be grouped if they share targets and sectors. Such groups are named options [2,6,8]. While options might provide a good summary of previous actions, their breadth makes extracting specific actions difficult and burdens transferability from a planning perspective. An example can be found in Table 2, where in the option *Resilient communities to flooding* we find several specific measures, like *Heighteniing dikes* or *Floating roads.* Then, each measure has a number of attached instruments which can facilitate or improve their implementation, like *Collecting high-quality data for flood recovery* or develop *Sustainable mobility plans in the cities*, and a number of SOIs which can be useful to quantify the outcomes of the measures.

**Table 2:** Hierarchy of options – measures – instruments – SOIs. Source: Authors.

Option	Measure	Instrument
Resilient communities to flooding	Heightening dikes	Flood management plan Collecting high-quality data for flood recovery Updating flood hazard maps Monitoring infrastructures
		**SOIs**
		Number of deaths, missing people, and persons affected by disaster per 100.000 people. Population exposed to flood risk Urban flood risk or areas exposed to flooding
	Floating roads	**Instrument**
		Flood management plan Sustainable mobility plans in the cities Include a buffer height for sea level rise when planning new developments
		**SOIs**
		Number of deaths, missing people, and persons affected by disaster per 100.000 people. Traffic accidents with victims (injuries and deaths) per 100.000 passengers Urban flood risk or areas exposed to flooding

**Fig. 2:**
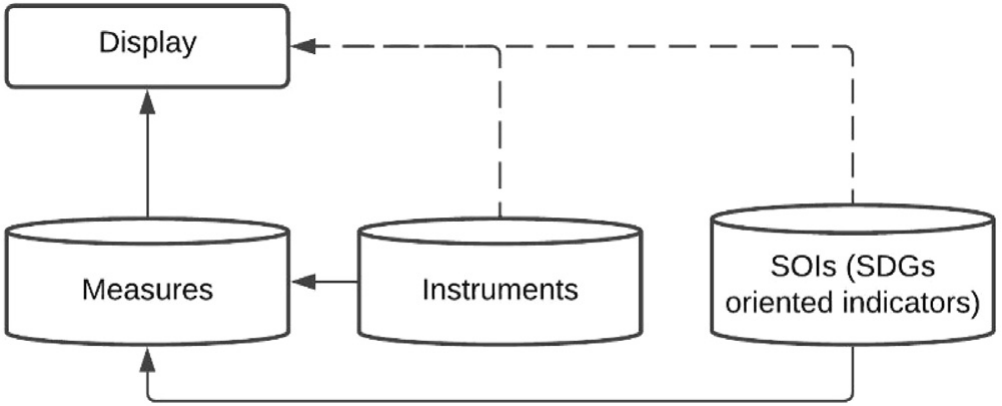
Generic structure of the database. Source: Authors.

Thus, our database [19] devotes itself to measures, as these are the minimum quantifiable climate action unit, and necessarily includes instruments and indicators for each. From a data point of view, the previous description can be translated into Fig. 2, which shows how instruments and indicators data are associated with each measure. The methods used to construct this relationship are detailed in the last portion of the Methods section.

### 2.2 Main data types

Two main types of data can be found within the datasheets: (1) continuous (quantitative) and (2) nominal (qualitative) (Table 3). We identified reliable sources for both data types in the compiled secondary sources but faced challenges for each.

**Table 3:** Types of data contained in the database and how they are displayed. Source: Authors.

Data	Type of data	Pre-processed data found	Post-processed data
Qualitative	Nominal	Descriptions and names	Summarised.
		Categories	Aligned with the SECAP categories.
Quantitative	Continuous	Single values	Value rounded to the closest whole number.
		More than one value	Average and rounded to the closest whole number.
		Ranges	Kept the range as in the original data.

For continuous data, we tried to maintain it in its original form. However, climate action implementation is highly context-dependent [2,6,8,9].This fact is highlighted by the differences in provided source values. To address this, we assigned value ranges or approximate average values using the raw data.

Nominal data presented a bigger challenge, as no alignment was found between the qualitative attributes within previous sources, which limits transferability and the harmonisation of data. On the contrary, systematic approaches as tools developed by international initiatives are crucial in developing Local Climate Plans [10,11]. Local Climate Plans are policy strategies and drafting specific actions to achieve climate goals and are crucial to reduce GHG emissions and adapt to climate change impacts. Therefore, we looked at one of the largest global climate initiatives with more than 11.600 signatories, the Covenant of Mayors, and used their template for developing Sustainable Energy and Climate Action Plans to establish a common framework to categorise the data. In such a manner, data might become transferable from our database to real climate planning practices.

### 2.3 Measures dataset

There are 188 mitigation measures, 191 adaptation measures, and 97 measures that can serve both types of response [19]. We defined 18 different variables according to existing scholarly climate planning literature. The selection of variables defining measures is based on (1) the importance of the gap between planning and implementation, according to the literature, and (2) the required data to provide in the SECAP templates. The 18 variables can be split into four data categories, depending on the information they provide (Table 4).

**Table 4:** Variables included in the measures’ dataset. Source: Authors.

Measure descriptors	Complementary items
ID of the measure	Associated instruments
Name of the measure^a^	
Description of the measure^a^	SDG's Oriented Indicators
Sources^a^	

Data for implementation	Attributes of the measure

Time for implementation^a^^,^^b^	Type of response^a^
Lifetime^a^^,^^b^	Main sector^a^
Implementation costs^a^^,^^b^	Mitigation sector^a^
Maintenance costs^a^^,^^b^	Complementary sectors
Origin of the measure^a^^,^^b^	Main hazard^a^
Stakeholders involved^a^^,^^b^	Affected hazard

a Variable transferable to SECAPs.

b Estimated values.

*Measures’ descriptors* give the basic information of the measure itself, such as their ID number or name (Table 5).

**Table 5:** Measures’ descriptors. Definitions. Source: Author.

Name	Description	Data type and categories
Name of the measure	Name given to the measure.	Nominal
Measure ID	ID assigned to a measure. Each measure has a unique ID.	Nominal
Description of the measure	Brief description of the measure.	Nominal
Sources	References, links, and other information sources used to fill the database, per measure.	Nominal

*Attributes of the measure* are related to the measure's target. After reviewing existing scholarly literature, previously built databases, and climate planning tools like the SECAPs, we concluded that there are four main attributes for measures: type of response (all), sector (SECAPs; Climate-ADAPT; RESCCUE; RESIN; EEA), and – in the case of adaptation – the hazard(s) addressed (RESCCUE; Climate-ADAPT; RESIN; SECAPs), or – in the case of mitigation –, mitigation sectors (SECAPs) (Table 6).

**Table 6:** Attributes of the measures. Definitions, categories and (quantity). Source: Author.

Name	Description	Data type, categories and (quantity)
Response type	It defines the goal of the measure. Measures can belong to three different response types, as the database integrates mitigation and adaptation measures. - Adaptation: The measures are used to cope with climate impacts. - Mitigation: The measure's ultimate goal is to reduce GHG emissions. - Adaptation & Mitigation: The measure can be used for both.	Nominal: - Adaptation (191) - Mitigation (188) - Adaptation & Mitigation (97)

Main sectors	The main sectors are related to the highest level of categorisation of the measures and refer to the field a measure addresses. The categories are built according to the SECAPs framework. There are two main sector types: mitigation sectors and vulnerable adaptation sectors. A measure can address more than one sector. Vulnerable adaptation sectors cover more economic, environmental, and social areas than mitigation sectors. In fact, some sectors serve both mitigation and adaptation. For example, Agriculture and Forestry is also important for mitigation [4], although not mentioned as mitigation sector in the SECAPs. Likewise, some mitigation measures, e.g. those related to tourism (like promoting local commerce), environment and biodiversity (creating parks and green areas), and education (any course related to shift into behaviour), also relate to adaptation sectors. Therefore, our database adopted the adaptation sectors as the standard measure categorisation.	Nominal: - All (2) - Buildings (86) - Transport (82) - Energy (112) - Water (156) - Waste (34) - Land Use Planning (29) - Agriculture & Forestry (53) - Environment & Biodiversity (54) - Health (2) - Civil Protection & Emergency (17) - Tourism (1) - Education (6) - ICT (Information & Communication Technologies) (2) - Others (-) - NA (-)
Mitigation sector	As the SECAPs require mitigation sectors for each measure, these are also included in the database as specific data for all mitigation measures.	Nominal: - All (2) - Municipal buildings & equipment/facilities (54) -Tertiary (non-municipal) buildings & equipment/facilities (46) - Residential buildings (41) - Industry (38) - Transport (62) - Waste (11) - Local Electricity Production (39) - Local Heat/Cold Production (15) - Others (64) - NA (103)
Hazards	Hazard categories follow the categorisation of hazards in the SECAPs, which are also mostly coincident with some international standards, like EM-DAT. A measure can address more than one hazard.	Nominal: - All (10) - Extreme heat (61) - Extreme cold (20) - Heavy precipitation (84) - Coastal flood (77) - Fluvial flood (75) - Sea level rise (48) - Droughts and water scarcity (40) - Storms (57) - Mass movements (9) - Wildfires (8) - Chemical change (-) - Biological hazards (6) - Others (2) - NA (199)

Identifying interactions between measures can increase their effectiveness [3]. As the implementation conditions of measures vary according to their local and regional context [3,4,6,10,12], rather than identifying individual and particular interactions between measures, previous literature showed that identifying the side effects of measures might be the path to follow [6,13,14]. Thus, we added a second tier of sectors and hazards, indicating the sectors and hazards with which each measure potentially has synergies (Table 7).

**Table 7:** Attributes of measures. Complementary sectors and affected hazards definitions. Source: Author.

Name	Description	Data type, categories and (quantity)
Complementary sectors	There are trade-offs between different sectors, as shown in the previous examples. Thus, the database provides other sectors that might be affected by implementing a measure, offering a broader comprehension of what a planner should consider when planning a certain action.	Nominal: - Buildings (16) - Transport (15) - Energy (85) - Water (46) - Waste (18) - Land Use Planning (25) - Agriculture & Forestry (16) - Environment & Biodiversity (72) - Health (45) - Civil Protection & Emergency (16) - Tourism (3) - Education (8) - ICT (Information & Communication Technologies) (1) - Others. (-) - NA (201)
Affected hazards	The hazards that might be affected in terms of frequency, intensity, or impact when the measure is applied but are not the main target of the measure. It is important to note that some mitigation measures might have synergies with certain hazards, which are not exclusive for adaptation in our database.	Nominal: - Extreme heat (31) - Extreme cold (18) - Heavy precipitation (10) - Coastal flood (13) - Fluvial flood (11) - Sea level rise (22) - Droughts and water scarcity (14) - Storms (7) - Mass movements (31) - Wildfires (12) - Chemical change (8) - Biological hazards (8) - Others (-) - NA (310)

**Fig. 3:**
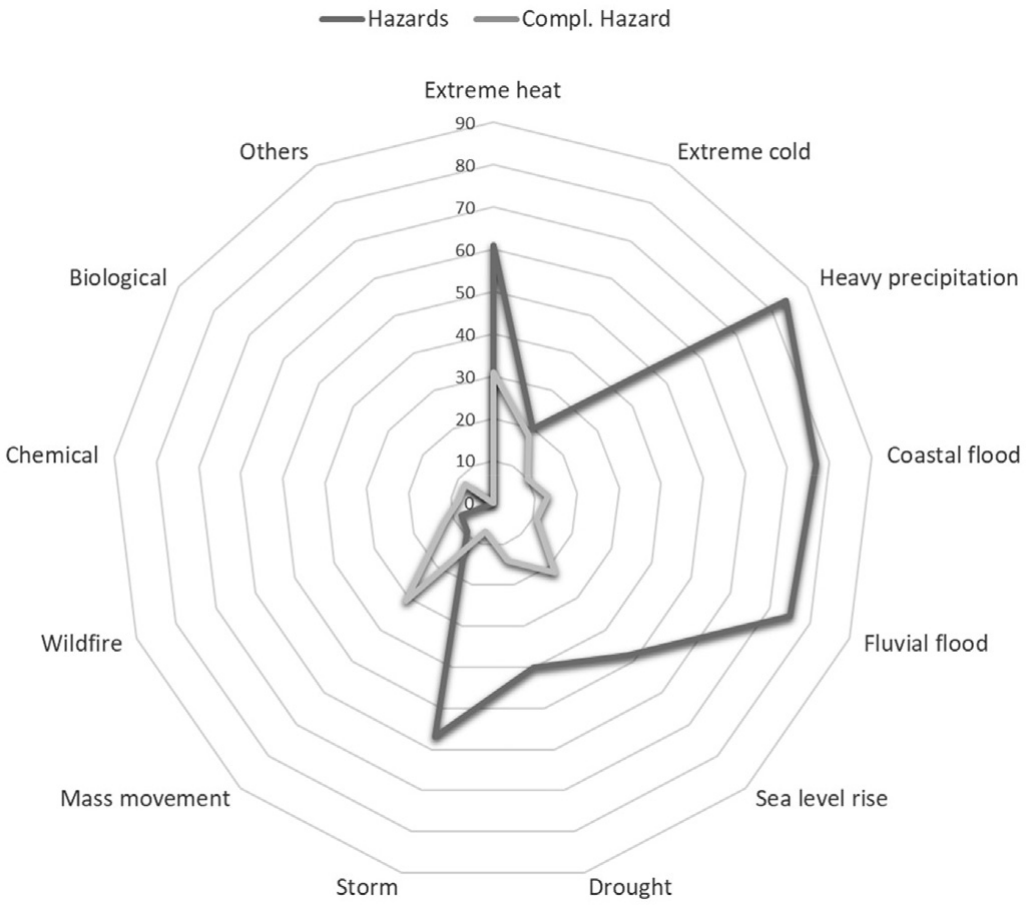
Measures addressing each hazard (dark grey) and impacting affected hazards (soft grey). Source: Authors.

**Fig. 4:**
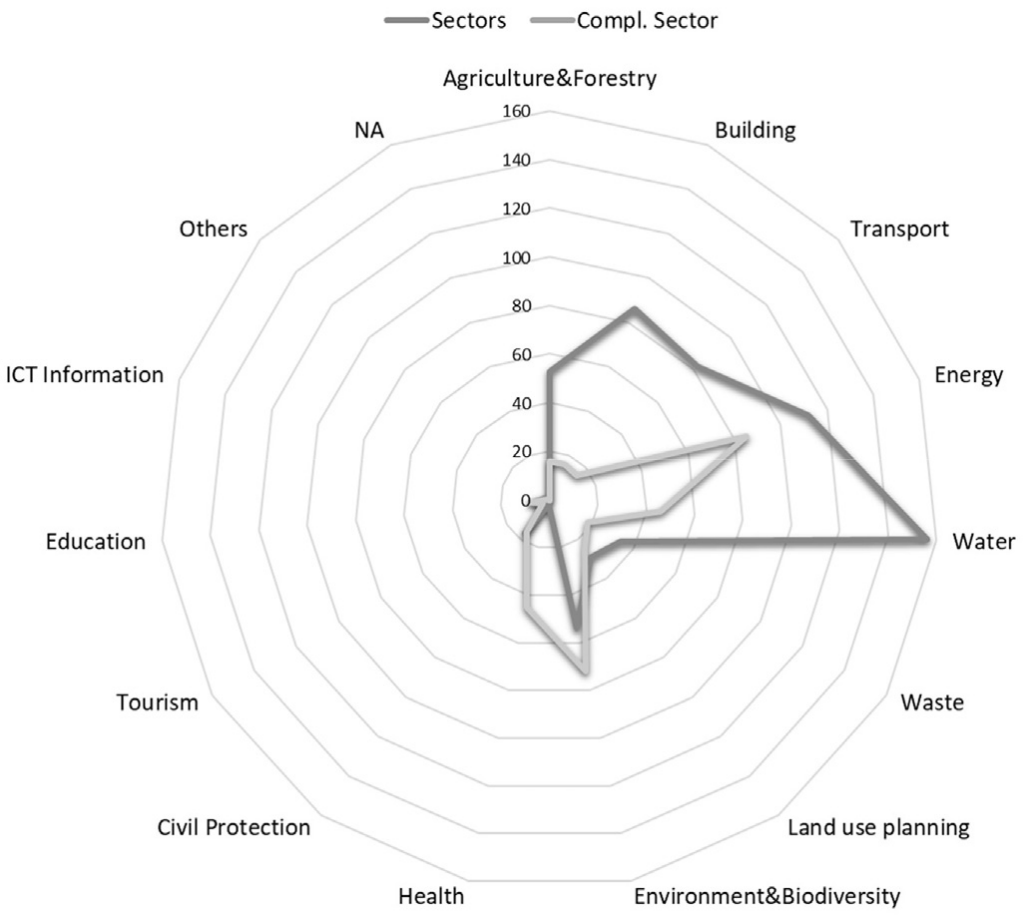
Measures addressing each sector (dark grey) and impacting complementary sectors (soft grey). Source: Authors.

The measures dataset [19] compiles 191 measures addressing only adaptation, 188 addressing mitigation, and 97 measures addressing both. Water is the most addressed sector by measures, with 156 measures, followed by energy, with 112. Adaptation measures are typically water-related, while mitigation is mostly connected to the transport and energy sector. In fact, the transport mitigation sector is the most addressed by mitigation measures with 62 measures. Regarding hazards, 84 measures address heavy precipitation, followed by coastal (77) and fluvial (75) flooding measures. In terms of complementary sectors, most measures have an impact on the energy sector (85) and on the environment and biodiversity sector (72). Even if only two measures are specifically addressing the health sector, this one can be impacted by 45 measures. As for the affected hazards, 31 measures might increase or reduce the effects of extreme heat, and 31 on mass movements, even though only eight measures directly address it (Fig. 3, Fig. 4).

*Data for implementation* refers to the data that might help close the gap between the planning and implementation stages and to develop better-quality plans. An appropriate planning scale [3,4], the proper consideration of implementation times and long-term planning [3,4], and the consideration of justice [5] increase the feasibility of climate actions. Moreover, identifying responsibility for the action and evaluating costs beforehand, among others, are points to be considered when developing high-quality plans [15] (Table 8).

**Table 8:** Data for implementation. Definitions. Source: Author.

Name	Description	Data type, categories and (quantity)
Implementation time	It is mandatory for the SECAPs and essential to developing a good quality plan. It is the period between the official publication of a measure into a plan and when the measure starts having an outcome on the targeted KPI.	Continuous

Measures’ Lifetime	An estimated value of a measure's lifetime helps planners account for long-term planning and avoid undesired obsolescence or lack of continuity in the measures.	Continuous

Origin of the action	Planning the measure at an appropriate scale will help reduce the possible gaps between the planning stage and its implementation. Scale is an essential issue regarding administrative boundaries and taking responsibility for implementation. Thus, this parameter is focused on the administrative scale and is aligned with the SECAPs’ “Origin of the action”.	Nominal: - Local authority (255) - Regional (109) - National (70) - Covenant coordinator or supporter (2) - Mixed (6) - Others (-) - NA (206)

Implementation costs	Installation costs refer to the development and implementation costs to make a measure operational. They are given as approximates or ranges using data available at the end of 2022. Double-checking the costs is important due to monetary value fluctuations and inflation rates. Besides, the greater availability of technology is expected to reduce the cost of some measures, and, when possible, the database provides an estimate of cost variation until 2050. Likewise for maintenance costs.	Continuous
Maintenance costs	Maintenance costs refer to those needed to keep the measure operating the closest possible to its original condition and losing the least effectiveness. Maintenance costs are typically calculated annually and increase over time due to obsolescence or price inflation.	Continuous

Stakeholders involved	Understanding which stakeholders and population groups are involved in a measure implementation helps reduce undesirable side effects and increase effectiveness. Combined with the administrative scale, it might help disentangle who takes responsibility for implementation. However, stakeholders and actors involved can vary depending on the region's regulations, policies, or instruments associated with the measures.	Nominal: - All (7) - National government and/or agencies (294) - Sub-national governments and/or agencies (272) - Business & Private sector (318) - Trade unions (18) - Academia (-) - Education sector (5) - NGOs (Non-governmental organisations) & Civil society (21) - Citizens (38) - NA (38)

**Fig. 5:**
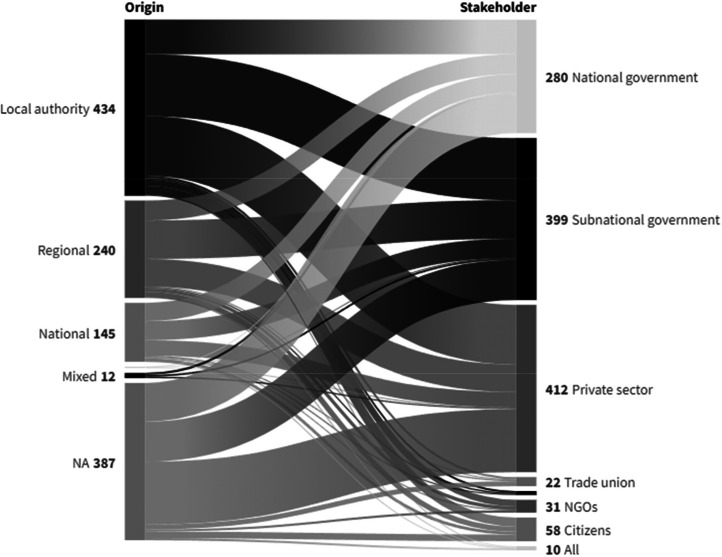
Number of connections between the scale initiating the measures – Origin of the measure – (left) and the stakeholders involved in implementation (right). Source: Authors.

According to the data collected, most of the measures need to be initiated at a local scale (Local Authority, 225). Meanwhile, looking at stakeholders involved in the implementation, the Business and private sector (318) and Sub-national governments (272) appear the most. As for any cost and time data, not all measures have data available, as it was subject to the availability of secondary data (Fig. 5).

*Complementary items* facilitate coherent, precise, and transferable climate plans. Moreover, they also help to include justice issues and synergies with SDGs from the perspectives measures. On the one hand, including associated instruments allows climate planners to assess mechanisms to define and modify the implementation of a measure and link complementary issues to it, like prioritising vulnerable groups. On the other hand, SOIs allow climate planners to understand how the outcome of a measure can be quantified while always remaining within the framework of the SDGs.

### 2.4 Instruments dataset

**Fig. 6:**
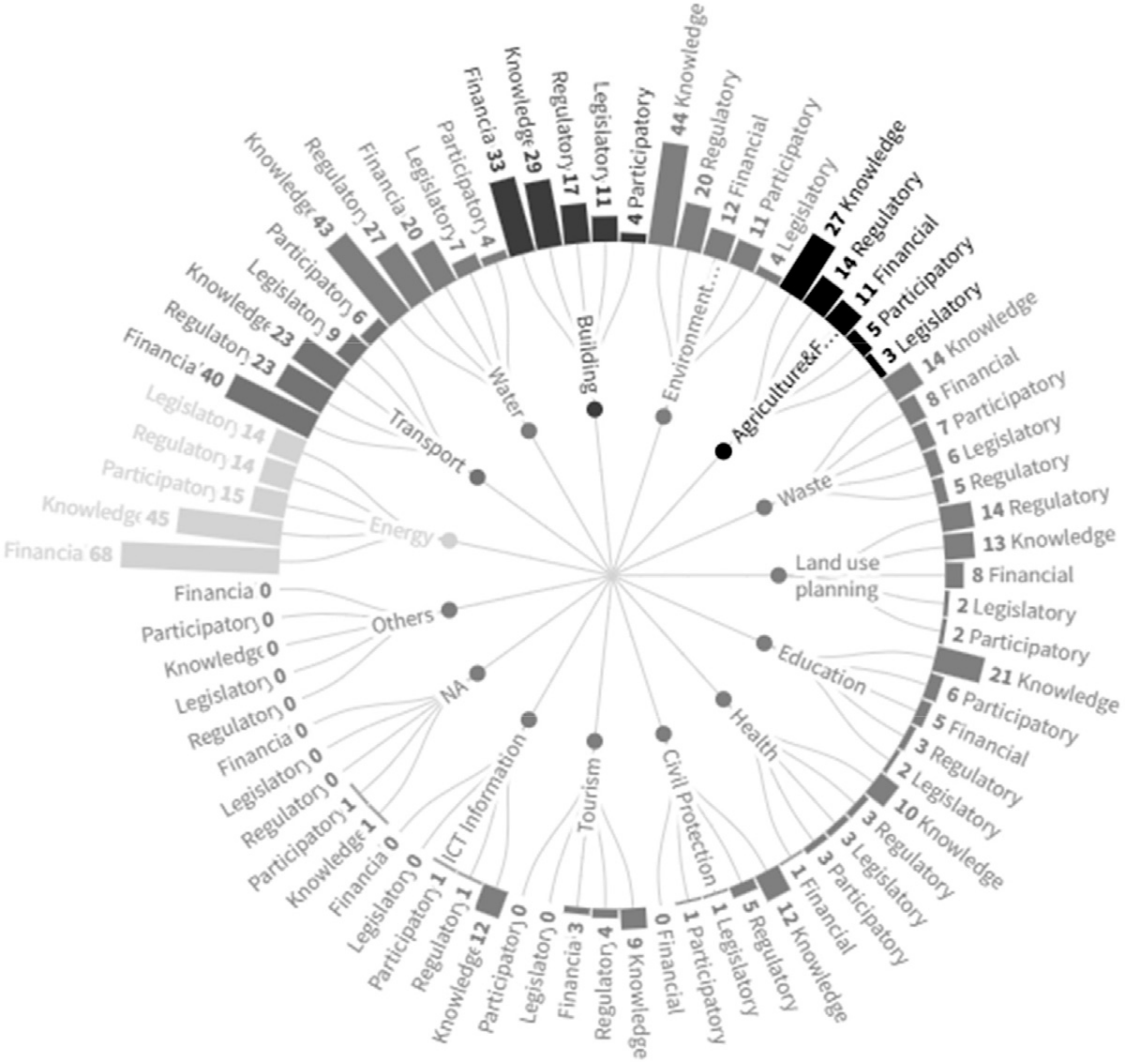
Number of instruments in each sector. Source: Authors.

While extracting measures from the previously mentioned sources, we detected 609 instruments that can be attached to measures. We assigned eight variables to define the instruments. Four of them are used to describe the instrument itself – name of instrument, instrument ID, description of the instrument, and sources –, while three are used to define its role and match with measures – sector, hazard, and origin of the instrument –, and one classifies them according to existing policy instruments framework – the type of instrument (Table 9, Fig. 6).

**Table 9:** Instrument datasheet structure. Source: Author.

Name	Description	Data type, categories and (quantity)
Name of the instrument	Name given to the instrument.	Nominal
Instrument ID	A unique ID assigned to an instrument.	Nominal
Description of the instrument	Brief description of the instrument.	Nominal
Sources	Per instrument, references, links, and other information sources are used to fill the database.	Nominal
Sectors	The main sectors are related to the highest level of categorisation of the instruments and refer to the field an instrument addresses.	Nominal: - All (82) - Buildings (88) - Transport (98) - Energy (147) - Water (98) - Waste (33) - Land Use Planning (34) - Agriculture & Forestry (58) - Environment & Biodiversity (82) - Health (18) - Civil Protection & Emergency (18) - Tourism (15) - Education (30) - ICT (Information & Communication Technologies) (10) - Others (-) - NA (-)
Hazards	Hazard categories will follow the categorisation of hazards in the SECAPs.	Nominal: - All (75) - Extreme heat (57) - Extreme cold (48) - Heavy precipitation (50) - Coastal flood (54) - Fluvial flood (52) - Sea level rise (48) - Droughts and water scarcity (32) - Storms (31) - Mass movements (8) - Wildfires (13) - Chemical change (2) - Biological hazards (5) - Others (2) - NA (245)
Origin of the instrument	The origin of the instrument shows the administrative scale at which an instrument is proposed.	Nominal: - All (10) - Local authority (384) - Regional (234) - National (286) - Covenant coordinator or supporter (12) - Mixed (7) - Others (-) - NA (50)
Type of instrument	Instruments are classified into five different types, which we adapted from existing frameworks according to our raw data [16,17].	Nominal - Legislative (49) - Regulatory (117) - Participatory (71) - Knowledge (267) - Financial (156)

### 2.5 Indicators dataset

**Fig. 7:**
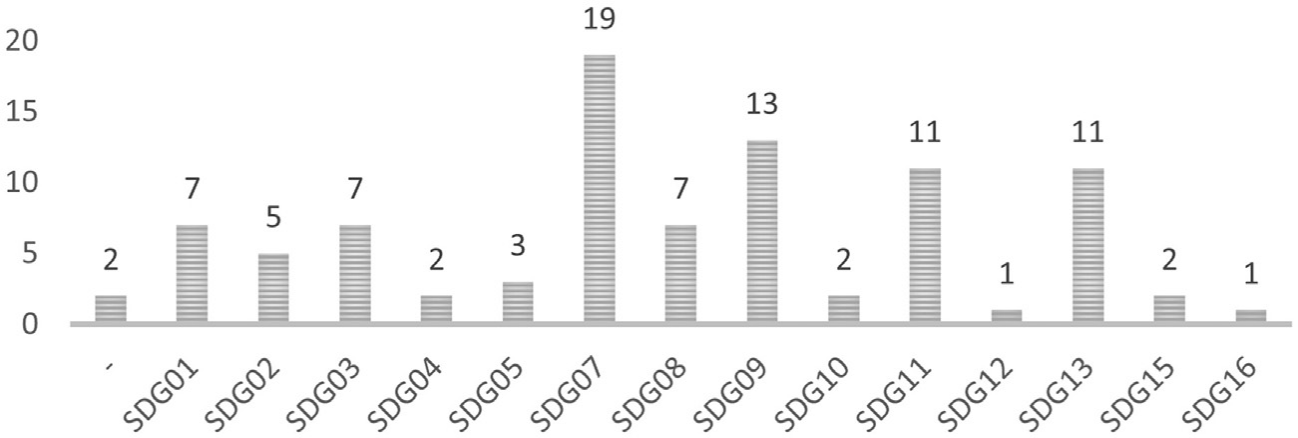
Number of indicators related to SDGs. Graph extracted from the data by Pascual & Ibañez Iralde, 2022.

Following the findings within the project LOCALISED [7] we extracted a list of 93 Sustainable Development Goals Oriented Indicators (SOIs). As defining quantifiable goals for climate actions helps improve a climate plan's quality [15] and identifying synergies with different Sustainable Development Goals might help increase the effectiveness of measures [3], we linked their work with our measures dataset to assign a set of SOIs to each measure. The links have been established qualitatively, according to the information obtained per indicator and the attributes and information acquired per each measure. In the end, each indicator has also attached data defined by us or extracted from the aforementioned publication (Table 10, Fig. 7).

**Table 10:** Indicator descriptors. Definitions. Source: Author.

Name	Description	Data type, categories and (quantity)
Name of the indicator	Name given to the indicator, extracted from LOCALISED D5.1 [7]	Nominal
Indicator ID	ID assigned to an indicator. Each indicator has a unique ID.	Nominal
Description of the indicator	Brief description of the indicator, extracted from LOCALISED D5.1 [7]	Nominal
Sources	References, links, and other information sources used to fill the database, per indicator, extracted from LOCALISED D5.1 [7]	Nominal
Sectors	The main sectors are related to the highest level of categorisation of the indicators and refer to the field an indicator addresses. The categories are built according to the SECAPs framework (Baseline Emission Inventory and Risks and Vulnerability Assessment) and coincide with the measures’ sectors.	Nominal:- All (9)- Buildings (19)- Transport (14)- Energy (25)- Water (7)- Waste (1)- Land Use Planning (3)- Agriculture & Forestry (11)- Environment & Biodiversity (4)- Health (7)- Civil Protection & Emergency (5)- Tourism (2)- Education (9)- ICT (Information & Communication Technologies) (7) - Others (-) - NA (18)
Hazards	Hazard categories will follow the categorisation of hazards in the SECAPs’ Risk and Vulnerability Assessments. The different categories coincide with the measures’ hazard categorisation.	Nominal: - All (1) - Extreme heat (4) - Extreme cold (3) - Heavy precipitation (4) - Coastal flood (3) - Fluvial flood (3) - Sea level rise (3) - Droughts and water scarcity (-) - Storms (3) - Mass movements (1) - Wildfires (-) - Chemical change (1) - Biological hazards (-) - Others (-) - NA (82)
Method	The method column briefly describes how the indicators can be obtained or calculated, extracted from LOCALISED D5.1 [7]	Nominal
Unit	The unit informs the user on which unit can quantify the indicator, extracted from LOCALISED D5.1 [7]	Nominal
Synergies with SDGs	Synergies with SDGs will indicate from which SDGs are indicators coming and which SDG will be positively affected by a positive outcome of the indicator, extracted from LOCALISED D5.1 [7]	Nominal: From SDG01 to SDG17

## 3 Experimental Design, Materials and Methods

**Fig. 8:**
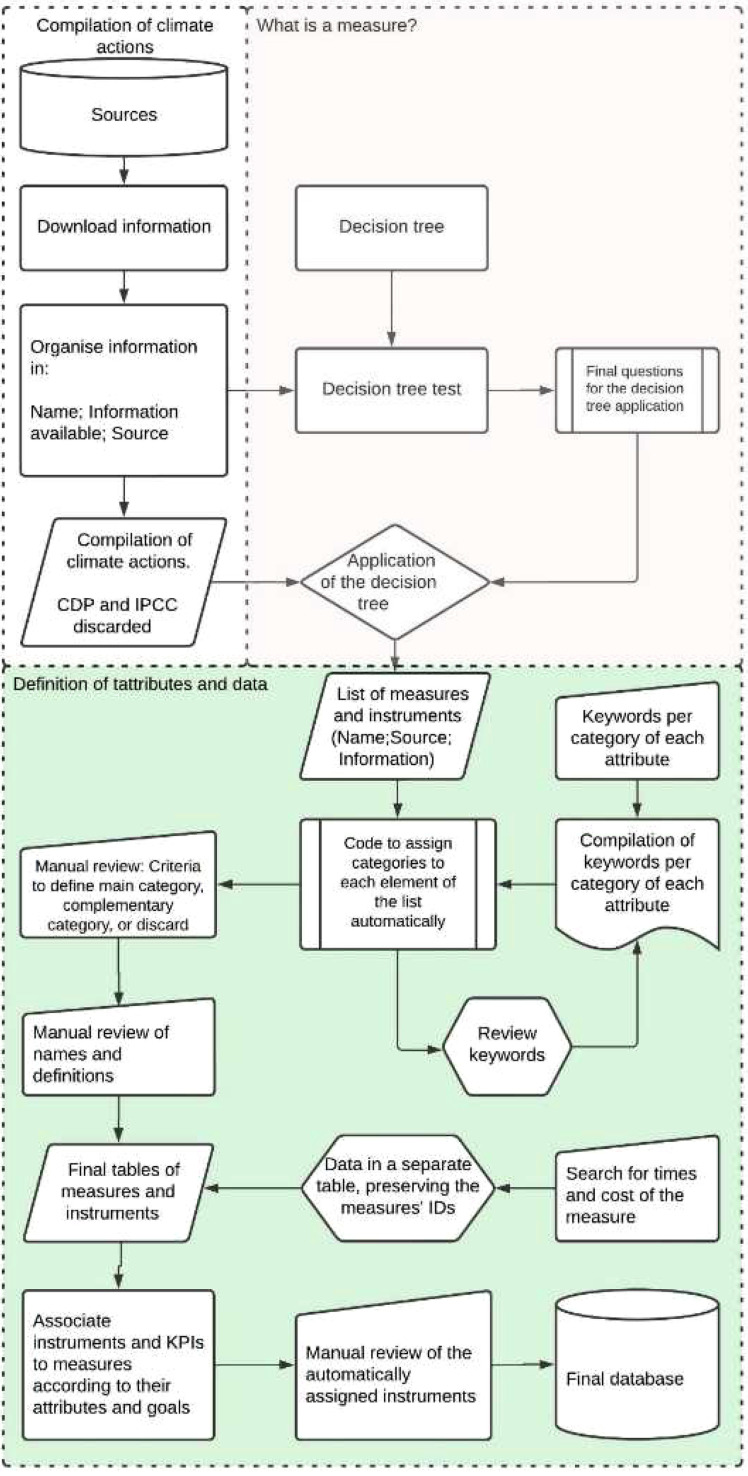
Process of construction of the database. Source: Author.

The process of building this new database can be summarised into three main steps: (1) compiling climate actions; (2) distinguishing climate actions according to their operational purposes; (3) reframing climate actions according to a standard planning practice framework (Fig. 8).

### 3.1 Compilation of climate actions

The first step of the research was to compile climate actions throughout the European context. To do so, we searched for established climate actions repositories to extract the data, with the following criteria: (1) the databases have an European scope; (2) the databases contain detailed definitions of specific climate actions; (3) the repositories should have been developed based on implemented climate actions; and (4) the information should be field-based. Table 11 summarises shortly the inclusion-exclusion table for the criteria:

**Table 11:** Inclusion and exclusion table of the criteria used to select the source repositories. Source: Authors.

Repository	European Scope(1)	Detailed definition (2)	Implemented actions (3)	Field-based information (4)	Included in database
Climate-ADAPT	Yes	Yes	Yes	Yes	Yes
RESIN	Yes	Yes	Yes	Yes	Yes
RESCCUE	Yes	Yes	Yes	Yes	Yes
6th AR IPCC	Worldwide	No	Yes	–	No
CDP Database	Worldwide, can be filtered	Yes	Yes	Yes	Yes
CLARITY	Yes	Yes	Yes	Yes	Yes
EEA MMPD	Yes	Yes	Yes	Yes	Yes
SuM4All	Yes	Yes	Yes	Yes	Yes
TEG Taxonomy Database	Yes	Yes	Yes	Yes	Yes
ENSU 2022	Yes	Yes	Yes	Yes	Yes
JRC Database [20]	Worldwide, can be filtered	Yes	Yes	Yes	Yes
OECD Data for Climate Action	Worldwide, can be filtered	No	Yes	Yes	No
Climate Policy database	Worldwide, can be filtered	No	Yes	Yes	No
Climate Watch Data	Worldwide, can be filtered	No	Yes	Yes	No

Even though all database compile implemented climate actions, not all of them contain detailed information of specific and transferable climate actions. For example, IPCC reports do not contain detailed information, nor are most of their actions specific enough; the OECD Database only focus on the monitoring of the actions, but does not disaggregate them at the detailed level of information; the Climate Policy database contains a broad selection of actions that are not specific nor described including the information we were aiming for. In the end, 10 repositories were used for the database construction. In case some information of the database was missing in the sources, the data was cross-checked with scholarly literature from Scopus and Web of Science and grey literature.

### 3.2 Distinction of climate actions according to the role in the planning process

To reduce climate actions to the same level of abstraction, we developed an original decision tree methodology to objectively discriminate between measures, instruments, and options. Based on the agreed definitions, we designed a decision tree consisting of five-binary questions. According to the consecutive answers to the questions, the method enables climate actions to be systematically designated as a measure, instrument, or option. As the information varies depending on the source and the type of item, the process should be conducted manually. Only measures and instruments are considered for inclusion in the database.

**Fig. 9:**
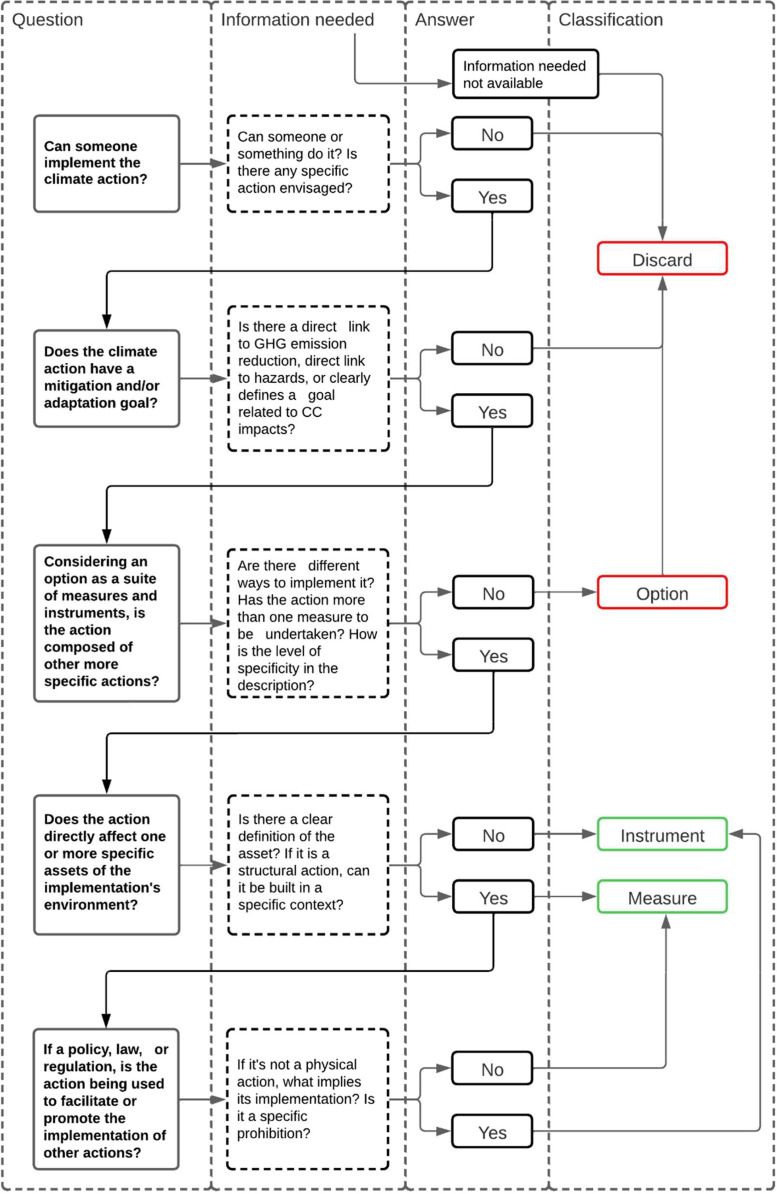
Decision tree procedure. Source: Authors.

To ensure the method's reliability, the decision tree was tested with five climate planning researchers of different backgrounds and institutions: the University of Twente, the Catalonia Institute for Energy Research, and the Potsdam Institute for Climate Impact Research. The test allowed us to refine the questions and understand the information needed to make the decision tree more robust (i.e., more objective). The subjects received two lists of the same ten randomly selected climate actions. First, they ran the decision tree only relying on the name of the climate action. Then, all available action information was provided and the test was rerun. No contact between the subjects was allowed. We departed from our hypothesis that the more agreement between answers, the more reliable and unbiased the method was. Results showed different levels of agreement according to the available information, but the agreement level increased significantly during the second part of the test. The process can be summarised in the following scheme (Fig. 9).

### 3.3 Harmonising climate actions

As explained, some nominal data needed to be re-categorised into predefined SECAP categories. To conduct this re-categorisation, we executed a semi-automatic keyword-matching method. The keywords were selected after consulting experts from 5 different institutions: University of Twente (UT), Österreichische Gesellschaft für Umwelt und Technik (ÖGUT), Forschungszentrum Jülich (FZJ), Catalonia Institute for Energy Research, and the Potsdam Institute for Climate Impact Research (IREC). Thanks to the different scientific backgrounds, expertise, language skills and cultural origins, a list of maximum 25 keywords was developed per category. The list was then filtered and processed as described in Table 13, and the final shortlist of keywords can be found in the Table S1 of the supplementary material. Following the matching method, we conducted a review process based on template coding to avoid mismatches and double-counting (Tables 12 and 14).

**Table 12:** Sources, types, and issues addressed by the 18 variables of the measures. Source: Author.

Variable	Are original categories coincident within the raw data sources?	Are original categories coincident with climate planning tools and practices (SECAP)?	Need to be re-categorised?
ID	Not applicable	Not applicable	Not applicable
Name of the measure	Not applicable	Not applicable	Not applicable
Definition of the measure	Not applicable	Not applicable	Not applicable
Source	Not applicable	Not applicable	Not applicable
Type of response	Yes	Yes	No
Sector	Some	Some	Yes
Complementary sector	–	No	Yes
Mitigation sector	No	No	Yes
Hazard	Some	Some	Yes
Affected hazard	–	No	Yes
Origin of the action	No	No	Yes
Stakeholders involved	No	No	Yes
Time for implementation	Not applicable	Not applicable	Not applicable
Lifetime	Not applicable	Not applicable	Not applicable
Installation cost	Not applicable	Not applicable	Not applicable
Maintenance cost	Not applicable	Not applicable	Not applicable
Associated instruments	Not applicable	Not applicable	Not applicable
Potential indicators	Not applicable	Not applicable	Not applicable

**Table 13:** Semi-automatic keyword matching process. Source: Author.

Step	Process	Outcome
1	Survey asking for keywords per each category per each variable to nine different climate planning institutions from different backgrounds and countries.	Maximum of 25 keywords per category
2	Run a matching algorithm, searching for the suggested keywords in the available raw data.	Categories assigned automatically to each measure, instrument, and indicator. Number of times the keyword has a match.
3	Analysis of the keywords with an anomalous number of hits.	Identification of conflicts and mismatches.
4	Discard contentless keywords – e.g., "management".	A final list of keywords.
5	Run a matching algorithm, searching for the final list of keywords in the available raw data.	Categories assigned automatically to each measure, instrument, and indicator.

**Table 14:** Criteria to identify the role of the category. Source: Author.

	The measure explicitly accounts for that, and the attribute is the principal in the definition.	The measure is indirectly related to the category; the attribute acts as a complement to the definition.	The word does not play any role in the definition, and the match was due to word misuse or a different semantic meaning.
Main sector	Accept.	Discard.	Discard.
Complementary sector	Discard.	Accept.	Discard.
Main hazard	Accept.	Discard.	Discard.
Affected hazard	Discard.	Accept.	Discard.
Origin of the action	Accept.	Discard.	Discard.
Stakeholders involved	Accept.	Discard.	Discard.
Mitigation sector	Accept.	Discard.	Discard.

### 3.4 Instruments and indicators to measures

**Fig. 10:**
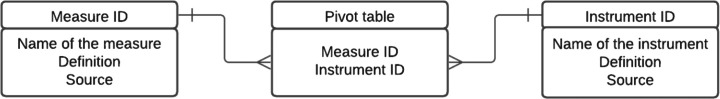
Many-to-many relations between measures and instruments. Source: Authors.

A single measure might have several instruments and indicators attached. Similarly, each instrument or indicator can be useful for multiple measures. Thus, measured data is related to instruments and SOIs data through a many-to-many relationship. Those relations can be explored using the coded datasheet “_Display”, which contains a simple exploring interface (Fig. 10).

Interlinkages have been qualitatively defined by comparing the Sectors, Hazards, and Origin of the measures variables from the instruments datasheet with the equivalent ones in the measures datasheet. During the process, it was noticed that some instruments were systematically referring to measures. In that case, instruments are automatically matched to the corresponding measure (Table 15).

**Table 15:** Assignment process of instruments and indicators to measures. Source: Author.

1. Categorisation of instruments			
Instrument	Main sector	[Other attributes]	Central topic
Grants for installing green roofs	Building; Environment&Biodiversity	[…]	Green roofs
2. Categorisation of indicators			
Indicator	Main sector	[Other attributes]	Central topic
Green area per inhabitant (m^2^)	Environment&Biodiversity; Land use planning	[…]	Green areas
3. Clustering instruments, indicators, and measures sharing the same attributes			
Main sector	[Other attributes] & Central topic	Element	Type
Building; Environment&Biodiversity	[…]& Green roofs	Grants for installing green roofs	Instrument
Environment&Biodiversity; Land use planning	[…]& Green	Green area per inhabitant (m2)	Indicator
Building; Environment&Biodiversity	[…]	Installing green roofs	Measure
Environment&Biodiversity; Land use planning	[…]& Park	Regulation for park construction	Instrument
Environment&Biodiversity; Land use planning	[…]	Building parks	Measure
4. Even if the relations with attributes might be diffuse, the central topic guided us to establish logical connections.			
Measure	Instrument	Indicator	Central topic
Installing green roof	Grants for installing green roofs	Green area per inhabitant (m2)	Green roof & Green
Building parks	Regulation for park construction	Green area per inhabitant (m2)	Green & Park

## Limitations

Limitations on the dataset are related to data availability and the context dependency of measures. First, there are several climate repositories online, and climate action is a trending field which is constantly being updated. The database was built on a selection of climate action repositories following certain criteria, using the most acknowledged and comprehensives ones as starting point. Nevertheless, small scale repositories, practitioners' compilations, on-going studies, and other less well-known databases that contain useful information might have been omitted.

Our data comes from extensive secondary data research. Thus, data on Measures and Basic Implementation are defined by their sources and cannot be modified. Cost and time data provide approximate values and ranges based on the specific sources from particular regions and case studies, while the Origin of the measure and Stakeholders involved may vary according to the implementation process characteristics. Moreover, when no reliable data regarding cost and time was found, then such data was excluded. While the database provides a framework and generic data to identify potential measures and actions, the Basic Implementation data should be checked before using it in practice. Nevertheless, the clear framework allows users and authors to constantly update the database with new values without compromising reliability and relevance over time.

## Ethics Statement

The authors have read and followed the ethical requirements for publication in Data in Brief, and confirm that the current work does not involve human subjects, animal experiments, or any data collected from social media platforms.

## CRediT authorship contribution statement

**Gerard Martínez Görbig:** Conceptualization, Methodology, Software, Validation, Formal analysis, Investigation, Data curation, Writing – original draft, Visualization. **Johannes Flacke:** Conceptualization, Validation, Writing – review & editing, Visualization. **Matthew Keller:** Conceptualization, Methodology, Validation, Writing – review & editing, Formal analysis, Investigation, Data curation. **Noah Pflugradt:** Conceptualization, Validation, Writing – review & editing. **Richard Sliuzas:** Validation, Supervision, Writing – review & editing. **Diana Reckien:** Conceptualization, Validation, Writing – review & editing, Supervision, Project administration, Funding acquisition.

## Data Availability

Integrated database on adaptation and mitigation measures in Europe (Original data) (Zenodo).

## Appendix Supplementary materials

Image, application 1
