# Supplementary material for: Fitting consistent knowledge into the planning process: An integrated database on adaptation and mitigation measures in Europe
Source: Data Brief. 2024 Jun 2;55:110580. doi: 10.1016/j.dib.2024.110580 (PMC11255512; doi:10.1016/j.dib.2024.110580)
Supplement: Supplementary file 1 [file mmc1.docx]

# SUPPLEMENTARY MATERIALS

Table S1 Final shortlist of keywords used for the methods. Source: Authors.

| Category | Label | Final list of keywords |
| --- | --- | --- |
| Sector | Building | Cooling, Roof, Heating, House, Insulation, Home, Window, Façade, Dwelling, Envelope, Architect, Incidence of the sun, Thermal protection, Sun protection systems, Cross ventilation, Housing, Built-Environment, Renovation, Ventilation |
|  | Transport | Transport, Road, Mobility, Motor, Street, Distance, Asphalt, Freight, Railway, Traffic Congestion, Highway, Transportation, Rush hour, Avenue, Car, Alley, modal split, Roadblock, Surface of roads, school paths, Bike lines, Pedestrian lanes, Maritime, Aviation, Modal Shift, Passenger cars, LDV, HDV, Shipping, Cycling, Public Transport, Transportation, Fuel, Driving |
|  | Energy | Energy, Heat, Power, Electricity, Energy supply, Lines, Grid, Cable, Energy poverty, Energy potential, Towers, Self-sufficiency, Power grid elements, Power Plant, Power Generation, Utility-Scale, Solar, Renewable, Fossil fuel, Energy grid |
|  | Water | Water, Flood, Sea, Drainage, River, Drought, Water supply, Aquifer, Withdrawals, Sanitation, Sewerage, Leakage, Chlorination, Private supply capture points, Water Shortage, Remote management (ICT), Conservation, Water Management, Rainfall, Hydrology, Irrigation |
|  | Waste | Waste, Treatment, Reuse, Dump, Recycle, Landfills, Leakage, Residual, Plastics, Recyclables, Collection itineraries, Food waste reduction, Food surplus, Recycling, Municipal Waste, Disposal, Circular Economy |
|  | Land use, planning | Land use, Landscape, Spatial planning, Urban planning, green space, Land cover, Greenery, Land planning, Territorial planning, Land classification, Permeable territory, Vulnerable infrastructures, Naturalize, Natural spaces, refreshment areas, Shading infrastructure, Seabed, Green Urban Area, Land Cover, Territory |
|  | Agriculture & Forestry | Tree, Agriculture, Forest, Wood, Crop, Forestry, Agricultural, Biomass, Fishing, Irrigation, Livestock, Agro, Irrigation systems, Horticulture, Cultivation, Agricultural products, Fire prevention, Charcoal, Urban agriculture, Local agriculture ,Organic agriculture, Forest management instruments, pest control system, Protection strips, Forestation, Afforestation, Deforestation, Farming, Forest Conservation, Soil Degradation, Carbon Sequestration |
|  | Environment & biodiversity | Environment, Pollution, Conservation, Biodiversit, Greenhouse gas, Green infrastructure, Reforestation, Preservation, Natural resources, Urban green, particular matter, Organic fertilizers, Recovery dunes, Preservation seabed, Biodivesity, Biosphere, Fauna, Flora, Species, Taxa, Ecosystem services, Ecosystem, GHG |
|  | Health | Health, Disease, Air pollution, Well-being, Respiratory, Death, Wellbeing, Hospital, Healthcare, Cardiovascular, Therap, Therapeutic forest, Healthy itineraries, Health council, Regular cleaning urban infrastructures, Shading, Solar protection, Hospitalization, Resilient Health System, Air-Quality, Health Authority, Therapeutic |
|  | Civil Protection & Emergency | Risk, Emergency, Safety, Warning, Tourism, Security, Relocation, Evacuation, Alarm, Crisis, Civil protection, Blackout, Number of drills, Hydrant ordinance, Response |
|  | Tourism | Tourism, Trip, Tourist, Visitor, Journey, Bioeconomy, European Charter for Sustainable Tourism, Percentage tourist tax, Company Badge, Eco certification, Eco-renewal actions  Travel, Smart tourism, Ecotourism, Hospitality,  Resort, Destination |
|  | Education | Formation, Education, Research, Training, School, Master, Learning, Seminar, Teacher, Symposium, University, Critical thinking, Literacy, Academic, Educated, Student, Higher Education |
|  | ICT Information & communication | Information, Communication, Digital, TV, Phone, Campaign, Drones, Social network, Intelligence, Advertisement, Digital twin, Communication Technology, ICT development index, IOT, Awareness, Message, Information Gap |
|  | All / Other | Scenario, Abatement, Industry |
| Hazard | Extreme heat | Wave, Cooling, High temperature, Extreme heat, Thermal comfort, Natural ventilation, Heat transfer, Melt, HVAC, Forced ventilation, Thermal perception, Heat Wave, heat accumulation, Intense Heat, Heat wave indices, Heat stress, Heat |
|  | Extreme cold | Wave, Heating, Radiation, Thermal comfort, Heat transfer, Extreme cold, Iced, Cold wave, Low temperature, HVAC, Stove, Frozen, Below zero, Dwelling performance, Thermal perception, Freeze, Cold Exposure, cold stress, Cold |
|  | Heavy precipitation | Rain, Storm, Precipitation, Raining, Heavy precipitation, Flash flood, Permeable areas, Drizzle, Sleet, Flooding occurrence, Water velocity, Water load, Sewer enlargement, Impermeable barriers, torrential rain, Heavy Rainfall |
|  | Coastal flood | Port, Sea, Wave, Coast, Current, Beach, Ocean, Tides, Coastal flood, Water depth, Permeable areas, Water velocity, Water load, Sewer enlargement, Impermeable barriers, Storm Surge, Flood inundation, Shoreline protection |
|  | Fluvial flood | River, Surge, Basin, Fluvial, Fluvial flood, Stream, Stuary, Water depth, Permeable areas, Water velocity, Water load, Sewer enlargement, Impermeable barriers, Upstream discharges, Flood Plain, Riverine flood |
|  | Sea level rise | Sea, Surge, Coast, Sea level rise, Beach, Coastal areas, Ocean, Tsunami, Water depth, Permeable areas, Water velocity, Water load, Sewer enlargement, Impermeable barriers, coastal dynamics, Sea level variability, Sea surface temperature |
|  | Droughts and water scarcity | Drought, Water scarcity, Evaporation, Reforestation, Green areas, Water stress, Arid*, Growth system capacity, Reuse of the gray waters, Rainwaters, water shortage, Drought indices |
|  | Storms | Storm, Tornado, Lightning, Floodings, Blizzard, Blackout, Gust, Monsoon, Tempest, Twister, Flooding occurrence, Water velocity, Extreme weather, Cyclone, Tropical storm, Hurricane, Dust storm |
|  | Mass movements | Landslide, Avalanche, Rockslide, Mass movements, Earthfall, Snowslide, Mudslide, Rain infiltration, Overburden, Slope stabilization, Migration, Displacement, Migrant, Refugee, Cliff, Slope, Sediments, Erosion |
|  | Wild fires | Combust, Burn, Wildfire, Carbon sink, Wild fires, Flame, Bonfire, Vegetation management, Fire adapted comunities, Forest fire, Fire risk, Burn Radius, Fire |
|  | Chemical change | Chemical, Gaseous, Substance, Chemical change, Chemical waste disposals, Algae Bloom, Chemical imbalance, PH Level |
|  | Biological Hazards | Organisms, Biologic, Substance, Biological Hazards, Bacteria, Disease, Parasite, Ebola |
|  | Other | Mortality, Disaster Risk, Adaptive Capacity |
| Origin of the action | Local authority | City, Local, District, Village, Municipality, Assembly, Local authority, City council, Town, Inclusive, Ensure compliance, Top down, Campaigns to raise awareness, Foresight of local authorities, Municipal, City authority, Local council |
|  | Covenant coordinator or supporter | Climate neutrality, Covenant coordinator, Covenant supporter, Climate contract, Campaigns to raise awareness, Locally adapted measures, Bottom-up management, Covenant Direction, Strategic guidance, Mayor, Covenant, Supporter, Coordinator, Facilitator |
|  | National | Nation, National, Government, Country, Minister, President, National Determined Contributions, NDC, Policiy, Top-down management, Campaigns to raise awareness, Federal, Nationwide, Federal Regulation |
|  | Regional | Region, Local, State, Regional, Municipal, Federal, Province, Autonomous community, CCAA, Federal states, SECAP, Implementation national policy, Regionally adapted measures, Top-down management, Campaigns to raise awareness, County |
|  | Mixed | Multilevel, Layered, Cross-cutting |
|  | Other | Oversight committee, Judicial, Regulatory Body, Entrepeneur, Enterprise |
| Stakeholders involved | National government or agencies | Government, State, Country, Minister, Expert, Policy maker, National government, National agency, President, Federal ministry, National socio-economic development, Planning and policy departments, Employment rates, Department, Bureau, Federal Agency, Federal Commission |
|  | Sub-national governments and or agencies | Region, Federal, Experts, Sub-national, Province, Sub-national agency, CCAA, Autonomous communities, Energy agency, Environmental agency, Regional socio-economic development, Planning and policy departments, Urban and rural analysis, Municipal, City Administration, Regional Council, State |
|  | Business and private sector | Industry, Business, Union, Private sector, Ceo, Non public, Sustainability department, Sustainable finance, Value-chain risks, Decision support model, Economic sector, Business sector |
|  | Trade unions | Deal, Market, Exchange, Transaction, Commerce, Labour, Trade unions, Chamber of commerce, Lobby, Sustainable development goals, Support social assistance program, Decision support model, Labor union, Worker association, Labor representative |
|  | Academia | School, Institute, Academia, University, Higher education, Identification of future problems, Development of effective solutions, Research oportunities, Academics, Researchers |
|  | Education sector | Formation, Inform, Education, School, Children, Learning, Student, Seminar, Education sector, Symposium, Training program, Climate change education, Climate framework guideline, Motivate children and youth, Teacher, Educator, School Administrator |
|  | NGOs and Civil society | NGO, Civil society, Sustainable development goals, Decision support model, Debate decision makers, Public actor, Community Organization, Alliance, CSO, Community, Communities |
|  | Citizens | Actors, Locals, Community, Household, Resident, Taxpayer, Civilian, Dweller, People, Townsman, Townswoman, Villager, Vulnerable groups, co-decide, Inclusive, Individuals, Involvement in activities, Climate Emergency Table, Actions taken, Campaigns, Training for professionals different sectors, Investments in schools |

Table S2 Description of .bas files that allow the functionality of the database. Source: Authors.

| File name | File description | Button correspondence |
| --- | --- | --- |
| SplitCellDynamicColumn.bas | The Macro allows to display the list of instruments attached to a particular measure as a separated list. | Update Instruments |
| SpliCellDynamicKPI.bas | The Macro allows to display the list of associated SOIs to a particular measure as a separated list. | Update KPI |
| CleanRange.bas | The Macro allows to clean the lists of instruments and SOIs that are currently being displayed for the selected measure. | CLEAN |
| CleanHazard.bas | The Macro allows to clean the selected Hazard from the filtering panel. | Clear Hazard |
| ClearOrigin.bas | The Macro allows to clean the selected Origin of the measure from the filtering panel. | Clear Origin |
| ClearResponse.bas | The Macro allows to clean the selected Type of Response from the filtering panel. | Clear Response |
| ClearSector.bas | The Macro allows to clean the selected Sector from the filtering panel. | Clear Sector |
